# Cholecystokinin inhibits inducible nitric oxide synthase expression in lipopolysaccharide-stimulated macrophages

**DOI:** 10.1186/cc12973

**Published:** 2013-11-05

**Authors:** Evelin C Carnio, Luiz GS Branco, Rafael S Saia

**Affiliations:** 1 Escola de Enfermagem de Ribeirão Preto - USP, Brazil; 2 Faculdade de Odontologia de Ribeirão Preto - USP, Brazil; 3 Faculdade de Medicina de Ribeirão Preto - USP, Brazil

## Background

Cholecystokinin (CCK) receptors are expressed in macrophages and are upregulated by inflammatory stimulus. *In vitro *and *in vivo *studies have demonstrated the ability of CCK to decrease the production of various proinflammatory cytokines. This study investigates the role of CCK on iNOS expression in lipopolysaccharide (LPS)-activated peritoneal macrophages, as well as the intracellular signaling pathways involved in affecting iNOS synthesis.

## Materials and methods

Experimental procedures were approved by the Comitê de Ética em Experimentação Animal - FMRP (protocol number 152/2009). Thioglicollate-elicited macrophages were obtained by peritoneal lavage and cultured in RPMI 1640 medium, 10% fetal bovine serum and antibiotics. Nuclear p65, cAMP and iNOS levels were determined using ELISA kits, CCK receptors and IκBα expression by western blot and nitrite by the Griess method. Data were compared by one-way ANOVA and significant differences obtained using the Tukey multiple variances *post hoc *test.

## Results

CCK reduced NO production attenuating iNOS mRNA expression (15.49 ± 10.80 vs. 113.16 ± 0.23 AU; *P *< 0.05) and protein formation. Furthermore, CCK inhibited the NF-κB pathway reducing IκBα degradation and minor p65-dependent translocation to the nucleus (543.78 ± 84.57 vs. 90.42 ± 9.13%, *P *< 0.05). Moreover, CCK restored the intracellular cAMP content activating the cAMP-protein kinase A (PKA) pathway, which resulted in a negative modulatory role on iNOS expression and nitrite production. In peritoneal macrophages, the CCK-1R expression was predominant and upregulated by LPS (0.61 ± 0.08 vs. 0.30 ± 0.09 AU; *P *< 0.05). The pharmacological studies confirmed that CCK-1R subtype is the major receptor responsible for the biological effects of CCK.

## Conclusions

These data suggest an anti-inflammatory role for the peptide CCK in modulating iNOS-derived NO synthesis, possibly controlling the macrophage hyper-activation through NF-κB, cAMP-PKA and CCK-1R pathways.

